# The struggle of Freemasonry and Islamic ideology in the twentieth century during colonialization in Indonesia

**DOI:** 10.1016/j.heliyon.2021.e08237

**Published:** 2021-10-22

**Authors:** Ajid Thohir, Dedi Supriadi, Mulyana, Faizal Arifin, Muhammad Andi Septiadi

**Affiliations:** a Department of History of Islamic Civilization, UIN Sunan Gunung Djati Bandung, Jl. AH Nasution No. 105, Bandung, 40614, West Java, Indonesia; b Department of Arabic Language and Literature, UIN Sunan Gunung Djati Bandung, Jl. AH Nasution No. 105, Bandung, 40614, West Java, Indonesia; c Department of Sufism and Psychotherapy, UIN Sunan Gunung Djati Bandung, Jl. AH Nasution No. 105, Bandung, 40614, West Java, Indonesia; d Department of History of Islamic Civilization, Sekolah Tinggi Ilmu Adab dan Budaya Islam Riyadul ’Ulum, Jl. Pesantren Condong, Setianagara, Kec. Cibeureum, Tasikmalaya, 46196, West Java, Indonesia; e Department of Political Science, UIN Sunan Gunung Djati Bandung, Jl. AH Nasution No. 105, Bandung, 40614, West Java, Indonesia

**Keywords:** Freemasonry, Colonialism, Dutch East Indies, *Priyayi*, Islamic movement

## Abstract

Dutch colonialization with a colonial pattern indirectly left the perpetuation of the *devide et impera* politics among indigenous elites in Indonesia. The use of the natives as colonizers had resulted in conflicts and increased competitiveness patterns between *priyayi* and *santri.* Consequently, the clash of religious ideology with the new aristocratic model (Dutch *priyayi*) was inevitable. Freemasonry's propaganda successfully recruited many indigenous elites and significantly contributed to Dutch Colonialism. This study was historical research with several stages: heuristics, criticism, interpretation, and historiography. Dutch Colonialism's upbringing was projected to successfully deal with the rise of *santri* organizations, such as Syarikat Islam (SI), Muhammadiyah, and Nahdlatul ‘Ulama (NU). These organizations aggressively revived resistance against the Dutch colonial in the 1920s. Freemasonry succeeded in clashing Javanese culture with Arabic (Islamic) culture to separate the indigenous elite from religious groups, considered radical and threatening Western colonialism. Freemasonry aimed to reject various forms of religious fanaticism and was an anti-religious dogma. The practice of colonialism and the role of Freemasonry has a common interest resulting in a mutually beneficial relationship.

## 1 Introduction

Colonialism in Indonesia resulted in several permanent political-cultural heritages, particularly in national life patterns in Indonesia (Peluso and Harwell, 2001; Young, 2018). Colonizers created large factions between religion and secularism as well as between religion and nationalism; this condition was frequently confronted to an extreme consequence. The dialogue among religion, secularism, and nationalism got stronger when the Freemasonry organization took a hidden role and infiltrated its sophistication. Thus, many historical roots in the dialogue are necessarily studied and explored. This study explored two objectives:

1. What are the structure and role of the Freemasonry organization in perpetuating Dutch colonization?
2. How are Dutch colonizers’ strategies to influence Indonesian indigenous elites who considered quite significant at that time.

Along with the Dutch colonial policy in governing their colony in Indonesian territory through a system of indirect roles, they ensured that the indigenous people supported them (Hidayat et al., 2020; Karpiel, 2001; Wibowo, 2019). This condition paved the way for Freemasonry players behind Dutch colonizers to freely build a superstructure of indigenous elites run secretly. As an underground organization, Freemasonry was not structurally and directly related to the Dutch Colonial Government. Still, its strategic vision had a common interest in assimilating Western ideologies into indigenous elites. They deradicalized religious politicians, especially students of an indigenous community.

The Freemasonry successfully performed propaganda to recruit many indigenous elites (Karpiel, 2001), who, in the future, face students organizations, such as Syarikat Islam, Muhammadiyah, and Nahdlatul Ulama. These organizations began to fight against the Dutch colonialists at that time. Freemasonry successfully clashed Javanese culture with Arabic (Islamic) culture to separate native elites from religious groups considered threatening Western colonization. This interest intersected with the goals of Freemasonry, which refused multiple forms of religious fanaticism and resisted religious dogmas. These shared interests produced a mutually beneficial relationship between the practice of colonialism and the role of Freemasonry. This study revealed that Dutch colonization infiltrated through Freemasonry as an association of certain social elites to gain more power and weaken religious values in political life in Indonesia.

## 2 Methodology

The historical research methodology can observe Freemasonry and Islamic ideologies in Indonesia. This method employs two historical sources: primary and secondary sources. Historical research consists of four stages: heuristics, criticism, interpretation, and historiography (Brundage, 2017).

Heuristics is an activity to search and find necessary sources. The success of heuristic probing depends on the researcher's knowledge of the sources and technical capacity to scrutinize the sources. The media of historical sources comprise of manuscripts, archives, documents, books, magazines, journals, newspapers, photos, dairies, and non-material sources, such as folklore and tradition. The characteristics of historical sources can be classified into the primary or secondary categories (Hirschler, 2013). The primary source refers to documents from official or authorized institutions, and the secondary sources refer to books and journal articles, mainly archived from the colonial period.

The evidence functioning as scientific research sources must be valued through external and internal criticisms. These criticisms refer to defining authenticity and credibility sources. External criticism is used to examine if the data is authentic (valid) or forged by investigating the date of the making, creators, and other aspects, such as letters, inscription styles, ink, writing tools, media, and language (Pace et al., 2011). Internal criticism is used to analyze whether the content is valid and credible (Mahmood, 2014). The criticism aims to select data towards building historical facts.

The interpretation of historical facts in the historical method is derived from archives, internet sources, relevant books, or direct interviews with actors. Moreover, this method is directly related to an event, object, research subject, or other people who know the investigated event. The interpretation stage compares one fact to another fact. The interpretation of a fact must be objective. If it is necessary, subjectivity is acceptable as long as it is rational and not emotional. Historical reconstruction must result in the truth or close to the truth. At this stage, analysis is conducted using theories adapted to research goals. The challenge and response theory by Arnold J. Toynbee simplifies the case investigation (Thohir and Sahidin, 2019a, Thohir and Sahidin, 2019b).

The last step is historiography to arrange the facts into a systematic or diachronic chronology and write them as a historical and scientifically trustworthy presentation (Kragh, 1987). Historiography is the summit of historical research and takes the form of a research report to recreate the totality of the historical facts by writing down the true event in the past and making some synthesis and analysis.

## 3 Results

### 3.1 History and missions of freemasonry in Indonesia

There is not much information about when the Freemasonry organization was formed because it was a secret organization. However, it is predicted that the movement for freedom of thought and anti-religious dogma has existed before the Middle Ages. Formally, the Freemasonry movement was founded in England in 1717 AD by merging four lodges into one grand lodge, called the Grand Lodge of England. Freemasonry then spread to mainland Europe, especially France, in the 1720s. In Netherland, the Grand Lodge of Nederland was founded in 1756 and later influenced the Dutch East Indies (Indonesia today). In Indonesia, Freemasonry was formally established in 1767 and 1769 with the declaration of “La Fidele Sincerity” and “La Vertueuse” lodges in Batavia (C. R. Hake, 1804).

Freemasonry's development and movement in the archipelago were strongly suspected of coinciding with the Dutch colonial agenda since the VOC era (Verenigde Oost-Indische Compagnie). The Freemasonry records between 1767-1917 describe the primary purposes of Freemasonry in Indonesia and can be traced. The purpose of Freemasonry was contained in its pamphlets written in Malay, Javanese (Latin and Javanese scripts), and Chinese (“De Ster in het Oosten”, Weltevreden, “La Constante et Fidele”, Semarang, “De Vriendschap,” 1917). The pamphlets massively invited natives to become members. Grand Lodge of Nederland recorded that Freemasonry members in the golden age of Dutch East Indies had reached 25 lodges with 1,500 members (Stevens, 2004). Freemasonry's aims and central visions were to improve behavior and intellectualism using true-life science. “De Ster in het Oosten”, Weltevreden, “La Constante et Fidele”, Semarang, “De Vriendschap,” (1917) proposed six principles that underlie Freemasonry. They are:

1. Respecting human dignity
2. Giving rights to anyone who will seek the perfection of conscience in their ways
3. Stipulating that each person must bear for himself regarding what will happen in his journey
4. Recognizing that all people are equal
5. Announcing brotherhood for all people
6. Stipulating that everyone must work earnestly towards the part of salvation for all

These principles were translated by Br. Rd. Ng. Sosro Hadikoesoemo.

The statement above explains that Freemasonry's goal to achieve heart purity and perfection can direct human behavior and intelligence, release a person to seek spiritual paths without being bound by specific religious rules, and provide equality between humanity and brotherhood. According to Kerr & Wright, Freemasonry bases its brotherhood on the bond of love, faith, and charity, and each member can communicate through various rituals and complex systems by elaborating ceremony and systems in the form of secret signs. Signs refer to sure passwords, for example, how to shake hands. Most of the Freemasonry rituals are based on moral teachings in the Old Testament illustrated or symbolized by the tools used by masons, namely the squares and compasses (Kerr and Wright, 2015).

During the colonial period, Freemasonry specifically chose *priyayi* as an indigenous elite and recruited them as a target in the Dutch East Indies. Moreover, Dutch colonial government selected *priyayi* as a partner to continue the government. *Priyayi* also became a liaison and implementer for policies and various regulations issued by the Dutch colonial government and the Kingdom of the Netherlands (Scherer, 1985). The *Gusti-Kawula* concept explains and provides an analysis of the relationship between a society as well as the *priyayi* and the Dutch. Moreover, this concept symbolized the balanced relationship between the king in the middle and his followers or people around him. However, this concept running for centuries was then changed after the arrival of the Dutch. The dutch made the relationship superior and subordinate, not circular. Therefore, the Dutch became *Gusti* for Javanese (Scherer, 1985).

However, the study by Scherer did not discuss the relationship between Freemasonry in the colonial era. In this era, the association supported priyayi's education and evolution of thought, which led to the process of transforming and adopting foreign (Western) values and thought into Javanese traditional values. Meanwhile, colonialism required an ideological transformation as part of legitimizing Western rules in the archipelago.

### 3.2 Genealogy, teachings, and structures of freemasonry

The interests of colonialists through social associations, such as Freemasonry, could be mutually beneficial for the two parties. Freemasonry recruited indigenous people with a mission of love for humanity because this action directed its members not to antagonize Europeans. This action was directly proportional to the colonial's goal that wanted a harmonious relationship between the indigenous government (*inlanders zaken*) as subordinates and the Dutch as a colonizer. In this case, Snouck Hurgronje was an adviser to the Dutch Colonial Government. He initiated and ensured that the educational process in society created two opposite poles. The first pole was *priyayi-abangan*, who was close to Sanskrit, Hinduism, Old Javanese, and New Javanese education as real politics. The second was *santri*, who was close to Arabic and Islamic education (Gobee and Andriaanse, 1990). Therefore, Snouck made and classified potential friends and opponents in the educational process that occurred in Dutch East Indies society. Freemasons seemingly benefitted these indigenous situations and conditions.

The Colonial Government aimed to direct the natives back to Javanese culture and ancient religion, counter religious doctrines and symbols, and separate them from Islam. Moreover, Freemasonry actively pursuaded Javaneses through subtle means.

1. Visiting old Javanese sites and not visiting Islamic historical sites in the archipelago
2. Giving lectures on ancient religions
3. Holding cultural activities supporting Freemasonry values
4. Inviting natives to contribute thoughts to Freemasonry

These activities were systematic attempts to campaign to the *priyayi* that “Islam” was very different and consistently hostile to Javanese culture.

Studying ancient religions was the primary theme for Freemasonry's teachings and became a symbol of opposition to formal religions. Some of the elements related to the symbolism in Freemasonry used various characters of ancient religions and mystical societies as well as various symbols of the builders or stoneworkers from the middle ages (A. C. Stevens, 1899). They learn and take lessons and meanings of spirituality from ancient religions in Asia, including Javanese and Indian spirituality. The emergence and development of Javanese spirituality or other local spirituality (Kebatinan) always drew the attention of the Dutch Colonial. As a counterweight to Islamic fanaticism among the *santri* community, Borobudur is seen as an ideal representation of Freemasonry buildings' (Perelaer, 1888). Borobudur remained one of the topics discussed in the Freemasonry meeting at the Deventer Lodge until 1917 (Kloosterman, 1917).

**Figure 1:**
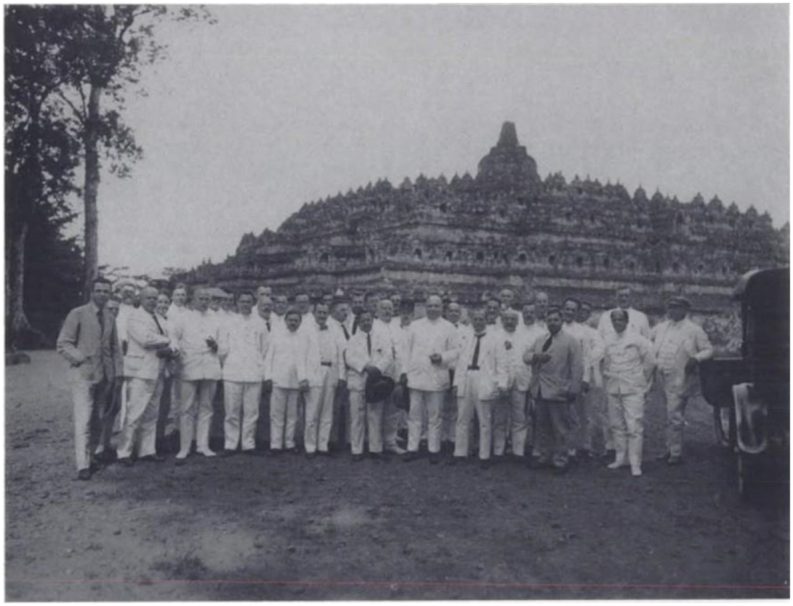
Members of the Mataram Loji travelling to Borobudur Temple in 1925 (Source: Stevens, 2004, Freemasonry and Society in the Dutch East Indies and Indonesia 1764–1962, p. 174).

An example of visiting an ancient Javanese non- Islamic site was Loji Mataram's activity to came to Borobudur Temple in 1925 as shown in Figure 1. One of the corridor walls of Borobudur shows the birth of Khrishna (the last incarnation of Vishnu) who was born from a representation of a virgin mother, from whom A Human-God will be born (Perelaer, 1888). This depiction gives the impression that the birth of Jesus from a holy virgin probably adopted this story. Therefore, visiting Borobudur not only showed the greatness of the Nusantara culture but also signified that Java has a high civilization exceeding Islamic or Catholic civilizations. During the visit, Huib van Mook, who later became Lieutenant Governor-General of the Dutch East Indies in the Dutch Military Aggression, wanted Indonesia to return to Dutch colonialism.

Perelaer conducted a symbolic interpretation of Israeli buildings (Solomon's Temple) and Hindu buildings, including Borobudur (Perelaer, 1888). From his point of view, he combined Jewish teachings from Solomon temple with Hindu teachings from Borobudur as a part of freedom to seek spiritual truth. Norwood Cyril explains that if we traced Freemasonry in the myth and legend aspects, its motives were related to the Crusaders with the Rosicrucians, the Roman Empire, Pharaohs (Pharaohs), Solomon temple, the Hanging Gardens of Babylonia (The Tower of Babel), and Noah's Ark (Cyril, 1934).

Freemasonry has a great legend about Hiram Abiff, who was later immortalized in the process of accepting a Freemasonry. The myth said that Hiram Abiff was killed by three people, and this tragedy became the ceremonial core of Freemasonry's admission. Hammer, square, and compass symbolized the killing of Hiram Abiff in a masonic ritual. The History Channel interviewed several Freemasons and Freemasonry organization researchers and revealed that these three objects symbolized three main enemies of Freemasonry, namely tyranny, ignorance, and fanaticism.

One of the three main enemies is fanaticism. Although every Freemason was allowed to pray to God according to his or her religion, they are not allowed to have fanaticism. Freemasonry viewed fanaticism as the absolute truth that came from religion and many ways. Robert Macoy quoted Johann Christian Gadicke's opinion in German's Freimaurer Lexicon (1818). Johann Christian Gadicke stated, “religious fanaticism cannot have any place in a Freemasons' lodge” (Macoy, 1873).

**Figure 2:**
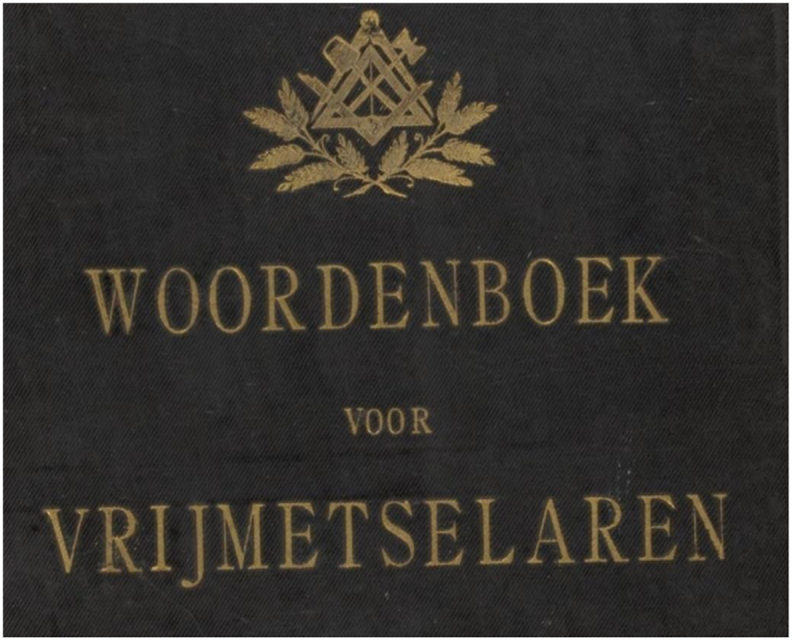
The hammer, square, and compass symbols in the front cover of A. S. Carpentier Alting's book entitled *Woordenboek voor Vrijmetselaren* (Source: A.S. Carpentier Alting, 1884, Woordenboek voor Vrijmetselaren, Haarlem: De Erven F. Born).

Figure 2 shows that the views and doctrines of Freemasonry did not allow too strong a belief in religion. This regulation was in contrast to religious teachings, which generally had the characteristics of fanaticism, especially to fundamental matters or the main basics. Islam, for example, requires its followers to believe wholeheartedly as stated in the creed that the truth comes from Allah. Therefore, the consequence of being a Muslim is rejecting other gods besides Allah, not acknowledging or justifying the existence of other gods.

### 3.3 Freemasonry and the interests of Dutch colony

The Dutch's colonial practices in the archipelago were performed through indirect rules; thus, they did not change the previous paternalistic bureaucratic and administrative systems (Hasan, 2018). In contrast, Britain performed direct rules in colonization in Asia by placing and involving Europeans directly in its governance structures. The Dutch always made indigenous elites an important part of strengthening their colonialism. Practically, this policy was carried out because manpower considering the vast expanse of the colony in the Dutch East Indies was inadequate or this policy was a strategic effort to be more recognized and accepted by colonized native people. Dutch colonists had full power at the government level, and they appointed indigenous elites to handed over the remaining power as regents, Patih, Wedana, Assistant Wedana, or Demang in rural areas. However, Dutch colonists had efforts to control the indigenous elites tightly. For example, Dutch colonists differentiated bureaucratic systems which made a regent and structures under him no longer had absolute autonomy to govern the region; they were limited by law with oversight from the structure of the central government of Dutch East Indies (Hasan, 2018).

Apart from the indigenous elites, the Dutch also made approaches through social organizations or associations that could benefit them. Freemasonry was the one that met Dutch expectations. Freemasonry had the same mission, especially in spreading liberalism and secularization. Therefore, the Dutch colony considered that Freemasonry could bridge the natives with Western secular thoughts and make natives accept the Dutch as “brothers” of civilization or oppos religious groups’ views. The establishment of some tribal organizations, such as the Boedi Oetomo, Jong Celebes, Jong Java, and Jong Soematra would certainly simplify the communication for the Dutch colonialists through Freemasonry. In other words, Freemasonry was considered successfully influencing the indigenous elites because it applied interaction among individuals as a routine activity of an organization with various symbols and their particular identities.

In reality, although a few Freemasons were involved in the Dutch power structure, the organization of Freemasonry did not have a direct structural-bureaucratic relationship with the Colonial Government in the Dutch East Indies. Freemasonry in the Dutch East Indies first appeared through the VOC Radermarcher in 1767. In other words, Freemasonry existed in the archipelago for the first time in 1767. This indicated that they had links to the VOC power structures, whose mission was trade. *Gedenkboek van de Vrijmetselarij in Nederlandsch Oost-Indië, 1767–1917* is a book published to commemorate the 150^th^ anniversary of founding Freemasonry in the Dutch East Indies. The book contains documentation and history of Freemasonry. Until this research was conducted, the book was still available as a collection of the Indonesian National Library.

The book, published in 1917, was resulted from a collaboration of three big lodges in the Dutch East Indies: the de Ster in het Oosten lodge in Batavia, the Constante et Fidele lodge in Semarang, and the de Vriendschap lodge in Surabaya. This book is one of the primary documents of the Fremasonry organization because it is the most comprehensive source to trace the history of Freemasonry in the Dutch East Indies.

**Figure 3:**
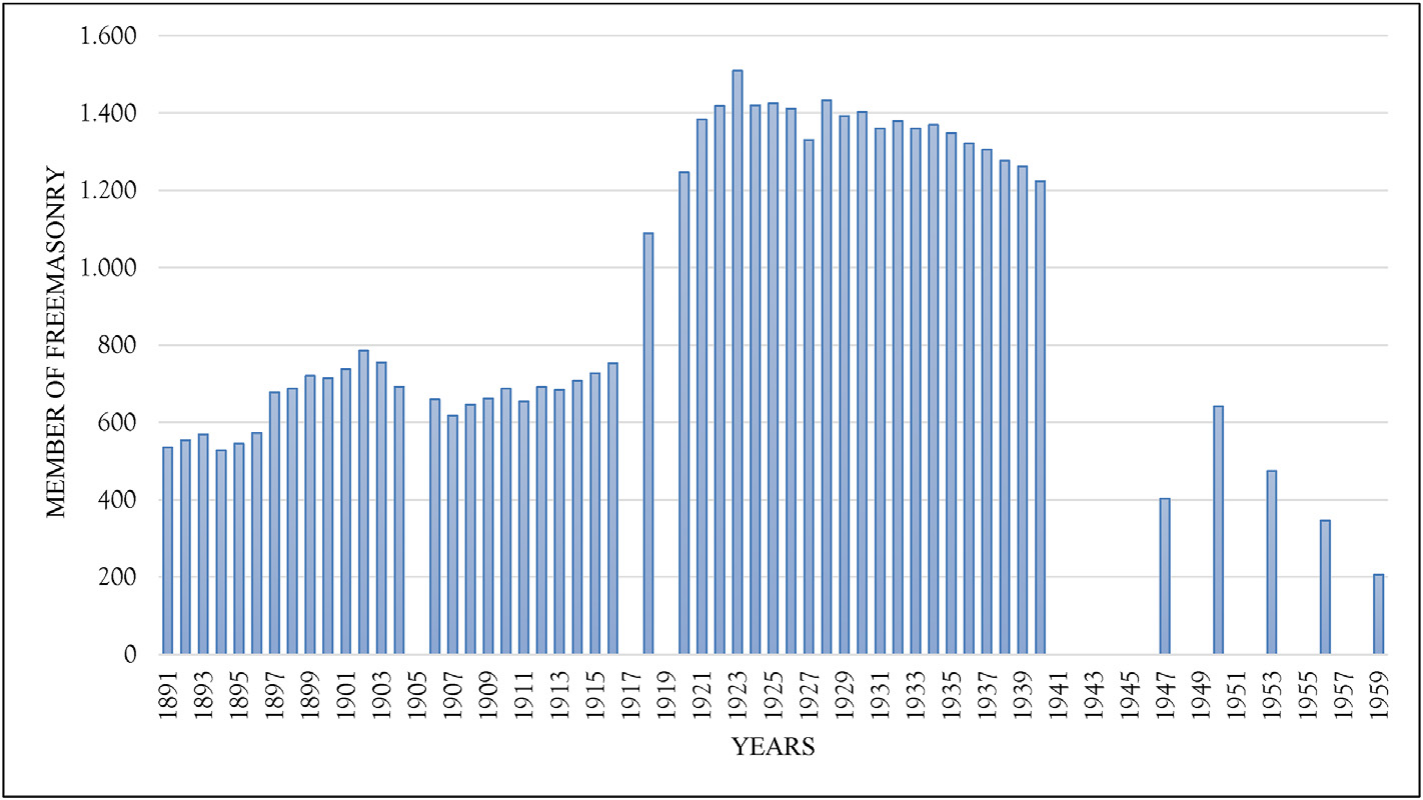
The total members of Freemasonry in the Dutch East Indies and Indonesia in 1891–1959.

Figure 3 shows a histogram referring to K. Hylkema's research on the membership of lodges in the Dutch East Indies. The histogram provides a comprehensive picture of the existence of lodges members, especially with the reality of the growth of lodges. Hylkema obtained several data of Freemasonry members from the 1800s to the 1940s. The development of Freemasonry members was inseparable from the appearance of various lodges in the Dutch East Indies (T. Stevens, 2004).

1. The La Constante et Fidele lodge was established in Semarang in 1801.
2. The De Vriendschap lodge was established in Surabaya in 1809.
3. The De Ster in het Oosten lodge was established in Batavia in 1837.
4. The Mata Hari lodge was established in Padang in 1858.
5. The Mataram lodge was established in Jogjakarta in 1870.
6. The Princes Frederik der Nederlanden lodge was established in Rembang in 1871.
7. The L'Union Frederic Royal lodge was established in 1872.
8. The Prins Frederik lodge was established in Kutaradja, Aceh, in 1880.
9. The Arbeid Adelt lodge was established in Makassar in 1882.
10. The Veritas lodge was established in Probolinggo in 1882.
11. The Deli lodge was established in Medan in 1888.
12. The Excelsior lodge was established in Buitenzorg (Bogor) in 1891.
13. The Tidar lodge was established in Magelang in 1891.
14. The St. Jan was established in Bandung in 1896.
15. The Fraternity lodge was established in Salatiga in 1896.
16. The Humanitas lodge was established in Tegal in 1896.
17. The Malang lodge was established in Malang in 1901.
18. The Blitar lodge was established in Blitar in 1906.
19. The Het Zuiderkruis lodge was established in Batavia in 1918.
20. The De Dageraad lodge was established in Kediri in 1918.
21. The De Broederketen lodge was reestablished in Batavia in 1919.
22. The Palembang lodge was established in Palembang in 1932.
23. The Serajoedal lodge was established in Purwokerto in 1933.
24. De Hoeksteen lodge was established in Sukabumi in 1933.
25. The lodge 'de Witte Roos' in 1948.

The emergence of these lodges simultaneously made the process of assimilation of European and indigenous cultures in the archipelago more intensive, especially for *priyayi* who were not students. Furthermore, the *priyayi* elites who joined the lodges proceeded to assimilate Western ideology.

**Figure 4:**
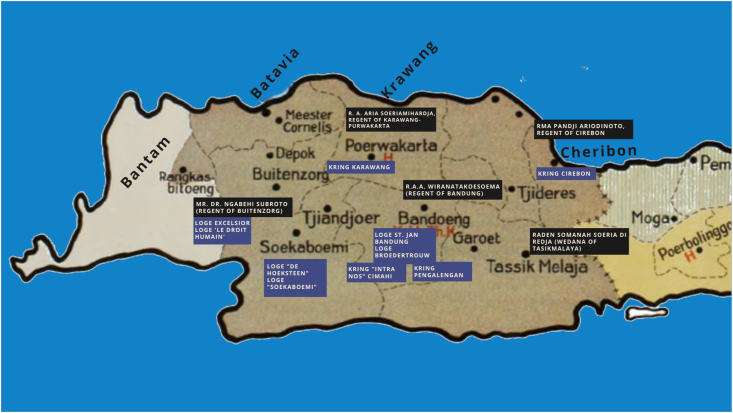
Names of lodges and *kring of Priyayi* Freemasons in several cities in Priangan (West Java).

Figure 4 shows a map of Priangan (West Java) in the Dutch Colonial era, several local rulers from *priyayi,* and locations of lodges in Priangan. Moreover Figure 4 denotes names of the *priyayi* who were Freemasons and describes the relationship between local political power and the secret organization.

## 4 Discussion

### 4.1 Assimilation movement and ideology for indigenous elites

Freemasonry had mass media, publishers, or printing presses to develop thought and convey their philosophy to people of the Dutch East Indies, especially indigenous *priyayi* elites who were the recruitment targets. There were some examples of works assimilating Javanese culture with Freemasonry teachings included the position of the highest leader of Freemasonry in the Dutch East Indies in 1914–1917. namely.

1. G. Andre de La Porte wrote *De Javaasche Beweging in het Teeken van de Vrijmetselarij* which means Javanese resurrection in Freemasonry (Artawijaya, 2010b).
2. In 1928, Pakoe Alam VII wrote a book whose cover was batik motif. The book entitled *Wat ik als Javaan voor geest en gemoed in de Vrijmetselarij heb gevonden* which means “What I found as a Javanese for spirit and soul in the Freemasonry”.
3. In 1930, Raden Sujono Tirtokusumo wrote an article entitling *De maconnerie onder de Javanen* which means Freemasonry among Javanese (Artawijaya, 2010a).

The two works of the indigenous elites showed a manifestation of the intellectual success of the *priyayi* who seriously responded to the positive assimilation process of Freemasonry with Javanese culture.

Snouck Hurgronje opines that the education process for natives was the only way to harmonize the relationship between indigenous people and European government elites in Java and elsewhere (Gobee and Andriaanse, 1990). In this context, Freemasonry's desired goal was to have native members who were lenient and not hostile to Europeans; this mission was directly proportional to the purpose of colonial education, which wanted a harmonious relationship between *priyayi* as the native government and the mission of Dutch colonialism. Therefore, there was no doubt that powerful frictions between the results of upbringing freemasonry and the anti-colonialism students would occur in the future and give a solid and negative stigma to the Dutch as infidels., Snouck Hurgronje in his assessment criticized Dr. Hazeu, who worked at the Ministry of Education, and argued that the educational process in society had a dichotomy of two opposing poles: the students and *priyayi*. Snouck Hurgronye stated:

“Naturally, on the one hand, the education of Sanskrit, Hinduism, Old Javanese, and New Javanese and all complementary languages is to make them more prominent. On the other hand, Arabic, Islam, etc. disregard these elements... (Gobee and Andriaanse, 1990).”

Snouck Hurgronje classified potential friends and potential opponents in the educational process that occurred in the Dutch East Indies society. *Priyayi*-*abangan* people were close to Sanskrit, Hinduism, and Javanese. and Meanwhile, *santri* referred to students who were “close” to Arabic and Islamic religious education. Snouck investigated the Aceh War. During this draining war, the life and property of the Dutch were materialized when the teachings of Islam against colonialism entered the minds of the Acehnese. Thus, this condition potentially threatened Dutch rule. The colonialists made Islamic religious and Arabic education for Javanese people, especially among *priyayi* and abangan. Moreover, they limited Islam and the Arabic language; thus, they only circulated in the students’ community.

Freemasons accustomed to meetings through typical styles and themes of Western culture and lifestyle. Various photos showed that their arrangements frequently used unique Freemasonry costumes, such as pants, white shirts, black coats, and accessories, such as medals or necklaces with Freemason symbols. This distinctive suit can be seen at various parties. For example, the management of the Mataram lodge was sitting with other members, such as Paku Alam VIII, Pangeran Soerjoatmodjo, Raden Soedjono Tirtokoesoemo, and R.M.A.A. Tjokroadikoesoemo, to commemorate Sint Jan day at the Mataram Lodge in 1934 (T. Stevens, 2004). Freemasons mentioned Paku Alam VIII as a figure who had many services for the Mataram lodge.

**Figure 5:**
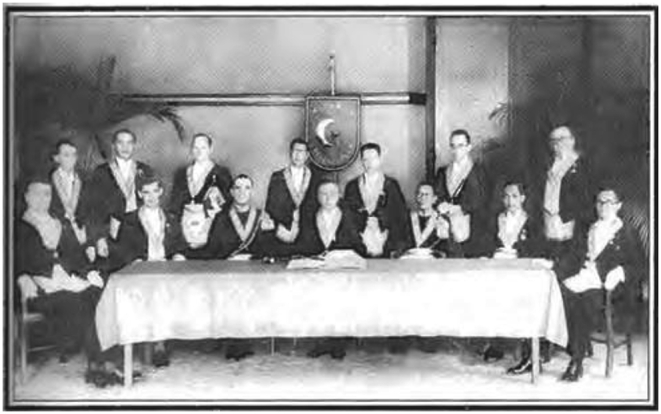
Celebration of Sint Jan Day at the Mataram lodge in 1934.

Figure 5 clearly describes the roles of the Javanese nobility, such as Pangeran Adipati Soerjoatmodjo (the Patih in the Paku Alam region), Raden Kamil (a member of the Volksraad in 1918–1924), and Soedjono Tirtokoesoemo (a translator and Patih in Blora). They assimilated Javanese and European culture through formal and non-formal meeting activities in the Freemasonry forum. They showed their identities, social language, and distinctive clothes to the native elites to influence native elites’ lifestyle and get recognition as more civilized, like Europeans.

They started with simple actions, such as dress styles and the language of their association. The indigenous Freemasonry members' thoughts and ideology began to be influenced. This condition agreed with Snouck Hurgronje, who divided two opposing poles: *priyayi-abangan* (Javanese culture) and *santri*. Freemasonry's activities were directed to the hostility of the two-pole thinking.

For example, Broderketen lodge in Batavia visited sites of Hindu civilization in 1922 to discredit Islam. One Freemason activist gave a speech saying, “According to Freemasons, Islam is a mixture of Arabic culture, Judaism, and Christianity. But Indonesia has its own culture. Arabic culture is not higher than Indonesia. Where do they have Borobudur and Mendut temples?” (Marzededeq, 2006). For Freemasonry, the return of humans to ancient religious teachings had important value because Freemasonry adhered to secularism hostile to Islam and Catholicism and prioritized the exploration for spiritual teachings from ancient wisdom and ancient religious civilizations. Moreover, they argued that beliefs in Egypt, Babylon, Mesopotamia, Ancient China, and Indonesia were from ancient Hinduism.

An article published in Suara Umum, a mass media owned by Boedi Oetomo under the guidance of dr. Soetomo in Surabaya, and quoted by A. Hassan in Al-Lisan Magazine number 24, (1938), stated that there was an inscription stating, “Digul is more important than Makkah. Throw away the Kaabaand make Demak as your Qibla!” This statement shows that the ideological assimilation of indigenous elites in Boedi Oetomo changed their religious view, especially Islam as the largest religion in Java. Therefore, people who took part in the Netherland administration and government, such as the intellectuals and *priyayi*, began to hate and hostile everything originating and referring to Islam (Hajriyanto, 2005).

Apart from Freemasonry, another mystical organization had the same roots of thought and interest in the Dutch East Indies; this organization was the Theosophical community. Freemasonry was founded in 1717, while the Theosophical organization was officially founded by Blavatsky in New York in 1875 (Nugraha, 2011; Syamsir et al., 2021). In a Theosophy website, Jay Kinney describes that the Theosophy founded by Helena Petrovna Blavatsky is a part of a Masonic sect that claims to be “Eastern” Masons and distinguishes themselves from Freemasonry, which is a “Western” Mason (Kinney, 2013). In America, although many Theosophy members are Freemasons, the two organizations do not have any conflict (Ashcraft, 2002).

De Visser Smits surveyed multiple memberships in the organizations and revealed that 600 of 3,878 freemasons in the Dutch East Indies mostly became members of other organizations from various social associations. This condition showed that as in America, Freemasonry members in the Indies were also members of other six organizations (T. Stevens, 2004). Freemasons in the Dutch East Indies possibly had a double membership. In other words, a person became a member of Freemasonry and Theosophy because the two organizations did not have any conflicting purposes, but they complimented another's deficiencies (T. Stevens, 2004). Another surprising fact was that both organizations had similar roots and characteristics. Therefore, it was reasonable to suspect that Freemasonry was an anti-opposition and resistance organization to Dutch colonialism.

Although Dutch people dominated the majority of indigenous members, most of them, who followed Theosophy or Freemasonry, considered that these movements united Javanese elites, Indo-European people, and Dutch people at that time. Moreover, they were very influential among many Boedi Oetomo members (Ricklefs, 2005). Boedi Oetomo was basically an institution that prioritized Javanese culture and education and rarely played an active political role. Therefore, it is natural that colonialism and Freemasonry influenced the thinking and behavior of indigenous elites in Boedi Oetomo. The influence of Freemasonry in Boedi Oetomo made many of its members become important figures in this *priyayi* organization.

Darban and Syakir examined the historical background of the establishment of Muhammadiyah and revealed that syncretism had many followers before the establishment of Muhammadiyah. However, Javanese society changed because this syncretist group had a strong organization recognized by its existence through Boedi Oetomo in the early 1900s (Darban, 2004). At that time, syncretism was a notion that all beliefs and religions were equally true. This symbolic ideology later became a factor in founding Muhammadiyah. Moreover, this study stated that t Muhammadiyah was important because its influence grew and its followers among Muslims multiplied. Adherents of the all-symbolism are an important aspect of Freemasonry teachings. Rene Guenon stated that Freemasonry is an organization that explored ancient wisdom, had full of symbolism, and performed worship rituals (Nurdi, 1991). Another expert stated that Freemasonry was a container of the breadth of traditional wisdom and had rich symbolism and rituals (Sakri, 2008).

**Figure 6:**
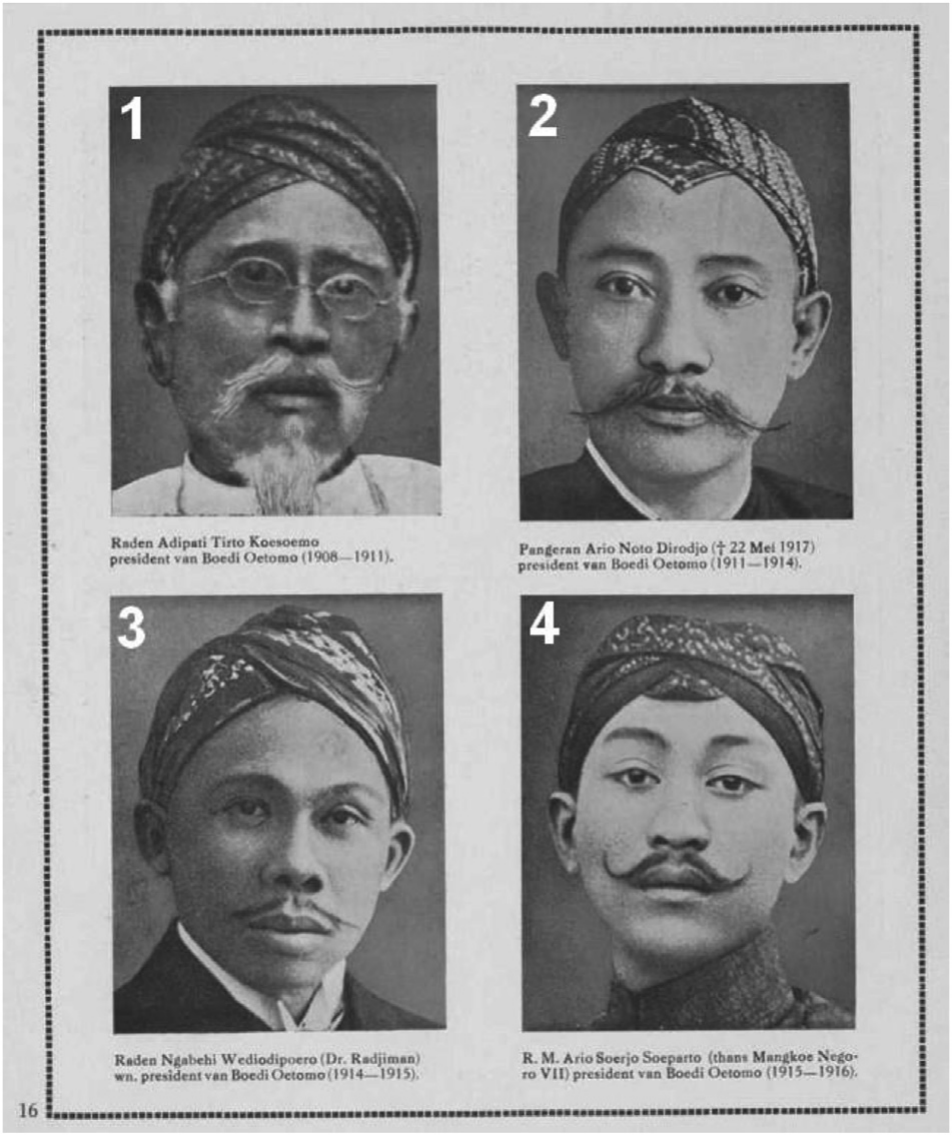
Figures of Boedi Oetomo in a ten-year memento book of Boedi Oetomo entitled Soembangsih Gedenkboek Boedi Oetomo (1908–1918).

Figure 6 shows an interesting point that four leaders of Boedi Oetomo were Freemasonry members, except for Number 4. Raden Adipati Tirto Koesoemo (Number 1) was the president of van Boedi Oetomo in 1908–1911 and was a Freemasonry member appointed at the Mataram lodge in 1895 (T. Stevens, 2004). Number 2 was Prince Ario Noto Dirodjo of the Pakualaman Palace who was the president of van Boedi Oetomo in 1911–1914, joined the Mataram lodge in 1887, and held various management positions in Freemasonry (T. Stevens, 2004). Prince Ario later became the chairman of Boedi Oetomo in 1911–1914. Number 3 was Raden Ngabehi Wediodipeoro (Dr. Radjiman), who was the president of van Boedi Oetomo in 1914–1915 and had been a Freemason since 1922 (T. Stevens, 2004; Van der Veur, 1976). Meanwhile, number 4 was R.M. Ario Soerjo Soeparto, who was later known as Mangkoe Negoro VII. He was the president of van Boedi Oetomo. He was not confirmed as a Freemasonry member but provided support for the Wederopbouw publication, a magazine influenced by theosophical thinking of mysticism in Freemasonry (Tollenaere, 1996).

Boedi Oetomo and Freemasonry organization hadit's a close relationship, as shown in the first Boedi Oetomo congress in Yogyakarta on October 3–4, 1908. This congress was initially planned to be held in the Freemasonry lodge, but because the lodge was used for another program, the congress was conducted in another place. The congress was actually held in logegebouw, a Freemasonry lodge building. People in Yogyakarta called it a “Satan House,” but at that time the house was only permitted to a team, who would hold a tentoonstelling (exhibition) of pictures (Soeharto and Ihsan, 1981).

Herman A.O. de Tollenaere explains a reason for why *priyayi* took part in theosophical activities and illustrates how many *priyayi* took part in Freemasonry because the Dutch Colonial Government's strategy was not to interfere with the social position of the previous Mataram power (Tollenaere, 1996). The Muslim *priyayi* still practiced various pre-Islamic traditions and pre-Hindu traditions. This phenomenon was similar to Freemasonry teachings that rejected official religions' practices but applied various symbols, rituals, and traditions in the pre-Christian and pre-Islamic era. On the other hand, the preaching (*da'wah*) of *santri* or Islamic organizations, such as Muhammadiyah, Al-Irsyad, Persis, etc. applied different pre-Islamic and pre-Hindu traditions and beliefs. Their using different pre-Islamic and pre-Hindu traditions and beliefs as a *da'wah* object indirectly, socially, and psychologically interfered with the sustainable values of the priyayi.

Another characteristic of the *priyayi* influenced by Freemasonry was shown in Sheikh Siti Jenar's teachings. It is plausible that the teachings of Theosophy and Freemasonry were mutually supported. Theosophical activists, generally Javanese elites who adhered to mysticism, considered Islam as an imported religion and incompatible with Javanese culture and identity (Marzededeq, 2006). A.D El Marzededeq, a researcher of the Freemasonry network in Indonesia, states that.

“The mystical association in Java was originated from the ideology of Sheikh Siti Jenar and increasingly supported the existence of Vrijmetselarij (Freemason). The Javanese elites embraced the concept of *wihdatul wujud* (the union of man with God) of Sheikh Siti Jenar and later became members of pure Theosophy-Freemasonry teachings or Javanese mysticism-mixed Theosophy-Freemasonry teachings.”

Javanese *priyayi* and figures who were Freemasonry or Theosophy members in the Dutch East Indies were frequently the masterminds of various harassments against Islam. For example, they argued that Boven Digul was better than Mecca, denounced the polygamy law, and considered Javanese (Gomojowo) or Kejawen religion better than Islam. Soewarni Pringgodigdo stated that polygamy was despicable and degrading to women, and if Indonesia wanted to be advanced and modern, it must abolish polygamy (Artawijaya, 2010a). Meanwhile, Homo Sun contended in the daily Soeara Oemoem that the pilgrimage only harmed Muslims, and *hajj* people (pilgrims) were not nobler than those exiled to Digul. Neutral religious circles shoed other unsympathetic attitudes recorded in the dialogue between Agus Salim and Singgih in Timboel magazine (Dault, 2005).

Adhyaksa Dault noted several cases regarding the attitude of supporters of “religiously neutral” nationalism. Boedi Oetomo positioned Islam and Muslims as contained in Djawi Hisworo Incident (1918), Kitab Darmogandul (1918), interview with Soetomo in the Indische Courant (1928), Timboel magazine (1929), and writings in Bangoen and Soeara Oemoem magazines (1930); Boedi Oetomo's position essentially opposed the idea of Islamic groups and insulted Islamic teachings (Artawijaya, 2010a; Dault, 2005). Koran Djawi Hisworo, published in Solo in 1918, reported that the secular nationalist group issued writings of Marthodharsono and Djojodikromo, who insulted the Prophet Muhammad and called him an opium drunkard and sucker (Dault, 2005).

These insults were carried out consciously through writings in the mass media and lectures at their associations. These insults became more acute when Freemasonry or Theosophy members actively participating in Boedi Oetomo organization clashed with activists of Syarikat Islam. MC Ricklefs, a Professor of Indonesian history from Australia, argued that in the 1900s Theosophy created intellectual movements, which were clearly anti-Islamic (Ricklefs, 2005). Ricklefs argued more clearly:

“Such movements link the bad situation in Java with the spread of Islam and try to restore the situation by unifying *budi* (Western a scientific perspective referred to “intellect”) and *buda* (Javanese culture before the entry of Islam). At the same time, Muslims began to conclude the need to revive the spirit and purify Islam. Thus, in the twentieth century, differences between two opposing ideologies emerged as the basis for national rejuvenation in Java.”

The clash of the two ideologies and the influence of colonialism in the perspectives of some Javanese *priyayi* groups occurred around 1900. This anti-Islamic tendency was near related to the interests of colonialism. Judging from the representation of members, Syarikat Islam (SI) indirectly faced Boedi Oetomo, who represented the *priyayi* and of which some members were Freemasons. The discrepancy was due to Boedi Oetomo's secularism by Dr. Radjiman Wediodipoera (1879–1952), known as Wedioningrat and a figure of Boedi Oetomo and Freemasonry (T. Stevens, 2004; Van der Veur, 1976). He criticized religious communities and predicted that SI was only a temporary movement of *hajj people*, *santri*, and ignorant people (Kuntowijoyo, 2003).

The influence and the side of the Netherlands on the priyayi who criticized SI also appeared in the dynamic ideological thoughts. For example, Dutch officials, such as Dr. Rinkes from the Kantoor voor Inlandsche Zaken, participated in ridiculing the *santri* by describing Haji Samanhudi as a gambler who frequently hang out with bad women, had multiple wives, and worked as a trader and loan shark “waking one morning and finding himself famous” (Kuntowijoyo, 2003). This statement showed efforts to discredit the image of the leader of the Islamic Trade Company (SDI) which later became the Islamic Company (SI). Consequently, the public and especially the Muslim *priyayi* did not follow the SI organization. Abu Hanifah, an Islamic movement figure argued that “incidentally, the aristocracy era, namely the feudals, showed an anti-Islamic union attitude. This condition was very encouraging to the colonial government” (Wiradipradja and Yahya, 2005). This statement indicated that one of the toughest challenges came from the *pangreh praja*, who was influenced by Freemasonry thinking. The Regents and other officials of Indonesia who served in the Dutch government seemed really afraid of losing their facilities if the Islamic Union became strong. Syarikat Islam movement generated a strong motivation for *santri* towards natives to have equal honor to other groups and nations.

Initially, *priyayi* and Dutch officials did not realize that SI would undergo a quite radical metamorphosis, as happened in 1914 when Tjokroaminoto's leadership gave an ideological flavour saying, “*de Islam is de godsdienst can de armen en de verdrukten*” which means Islam is a religion for the poor and oppressed people (Kuntowijoyo, 2003). The discrepancy occurred when the anti-colonialism ideology of SI began in 1915. In 1918 the anti-Feudalism attitude of SI was healthy towards the Javanese *priyayi*, especially shown by the second marriage of Pakubuwono X, who used traditional marriage, in terms of language, legal ceremony, and religious ceremony, in Solo. This anti-colonialism and anti-Feudalism attitude represented the union Islam ideology as one of the most phenomenal Islamic movements at that time. Moreover, the attitude simultaneously faced two super-ordinate powers: the *priyayi* and the Dutch Colonial Government.

Pradipto Niwandhono asserted that the first period of Boedi Oetomo (1908–1926) was the “association” period marked by the absorption of Western ideas in the world of the Indonesian movement (Niwandhono, 2014). During this period, organizations emerged by combining indigenous and western groups. Moreover, ideological assimilation was an inseparable part of the association process, Freemasonry and Colonialism especially influenced indigenous elites' thoughts. Governor-General van Heutsz, for example, welcomed Boedi Oetomo as he previously appreciated the publication of Bintang Hindia as a sign of the success of his desired ethical policy, namely creating a moderate progressive indigenous organization controlled by officials with a colonial perspective (Ricklefs, 2005). The Dutch colonial government's responses and appreciations resulted in many movements of dissatisfied people; thus, they suspected Boedi Oetomo as an effort to lull the natives from resisting and opposing colonialism. This attitude of suspicion was very well-founded because many Boedi Oetomo figures came from *priyayi* with close ties to the Freemasons and the Dutch colonial government. Raden Adipati Tirto Koesoemo, the Regent of Karanganyar, became a member of the Mataram lodge in 1895 and later became the first chairman of Boedi Oetomo (T. Stevens, 2004).

### 4.2 *Priyayi* membership and infiltration in freemasonry

Ricklefs illustrated the most prominent theosophists from the *priyayi* were Pangeran Pakualam VII, who ruled Yogyakarta from 1903 to 1938, and Susuhunan Pakubuwana XI, who ruled Surakarta from 1939 to 1944 (Ricklefs, 2005). It should be suspected that Belada infiltrated the centers of power of indigenous elites, especially the Javanese sultanates that were under the influence of Dutch East Indies colonialism through Freemasonry or Javanese Theosophy. The grandson of Paku Alam VII, R.A. Pandji Tjokronegoro, was also a Freemason because he celebrated his 50^th^ birthday as a Freemason in 1908 (T. Stevens, 2004).

The Dutch colonial gave the honorary title “Raden” to those who did not come from the Javanese sultanate family. This title gave a similar dignity symbol to the symbol of the royal family along with its unquestioned colonial loyalty. R.A. Pandji Tjokronegoro became a head of the Paku Alam family in 1908 and 1938, and a regent of Surabaya. Raden Sujono Tirtokusnomo became Patih of Wonosobo and Blora and was previously a member of the Mataram lodge in 1925 when he was a teenager studying with Paku Alam VII.

Another freemasonry from *priyayi* was Raden Mas Adipati Ario Poerbo Hadiningrat, a regent of Semarang and Salatiga. His opinion on the involvement of a Muslim to follow Freemasonry was quite interesting. He argued that there was certainly no incentive for people who were genuinely Muslim to become a Freemasonry member (T. Stevens, 2004). This statement was recorded in a small book with a batik-patterned cover published by Pakoe Alam VII in 1928 and entitled *Wat ik als Javaan voor geest en gemoed in de Vrijmetselarij heb gevonden* which means what I found as a Javanese for spirit and soul in Freemasonry. Freemasonry in the 19^th^ century continuously developed through lodges in various regions, such as Semarang, Surabaya, Batavia (Jakarta), Padang, Yogyakarta, Rembang, Solo, Kota Raja (Aceh), Makassar, Probolinggo, Medan, Buitenzorg (Bogor), Magelang, Bandung, Salatiga, Tegal, Malang, Blitar, Kediri, Palembang, Purwokerto, and Sukabumi (T. Stevens, 2004).

Outside the sultanate with Javanese cultures, such as West Java, some indigenous elite officials also became Freemasonry members. They were R. A. Aria Soeriamihardja as the Regent of Karawang and Mr. Dr. Ngabehi Subroto as the Mayor of Bogor (Buitenzorg). Japanese soldiers chased them in 1942 (T. Stevens, 2004). Van den Veur noted that several *priyayi* groups also became Freemasonry members (Van der Veur, 1976). They were R. Abas Soeria Nata Atmadja (the Regent of Serang and Cianjur), Rd. T. A. Achmad Probonegoro (the Regent of Batavia and previously the Regent of Semarang), R. M. A. P. Ariodinoto (the Regent of Cirebon, previously the Regent of Pemalang in 1908–1920), R. T. Aroeng Binang (the Regent of Kebumen), R. M. Darto Soegondo (the Wedana of Singosari), K. R. Ad. Djojonegoro B. K. O. A. A. (the Regent of Surakarta), Pg. A. A. Koesoemo Joedo (the Regent of Ponorogo), R. Marsoem (the Wedana of Parakan), R. Mohamad (the Wedana of Semarang), Notoadiprodjo (the Patih of Sidoarjo), P. A. A. Pakoe Alam, R. M. A. A. Poerbo Hadiningrat (the Regent of Semarang), R. M. T. A. Poernomo Hadiningrat (the Regent of Brebes, previously the Regent of Boyolali), R. Prawata (the Assistant of Wedana Yogyakarta and the Patih of Banjarnegara), R. Said Prawirasastro (the Wedana of Sidoarjo), R. M. Ng. Sarwoko Mangoenkoesoemo (the Secretary of Mangkunegaran Solo and the Regent-Patih of Mangkunegaran Solo), Mas Sewaka (the Patih of Indramayu), R. M. Soebali (the Wedana of Bumiayu and Purbolinggo), Rd. M. Soedjono (the Patih of Jepara), R. Soedjono Tirtokoesoemo (the Patih of Blora), R. A. A. Soejono (the Regent of Pasuruan), R. A. A. Soemitro Kolopaking Poerbonegoro (the Regent of Banjarnegara), Rd. Soeprapto (the Wedana of Wiradesa), Ir. R. M. P. Soerachman Tjokroadisoeria (the Regent of Serang), R. A. A. Soeri Mihardja (the Regent of Karawang and Purwakarta), R. Soerjo (the Wedana of Kertesono), R. T. A. A. Soerjo (the Regent of Pekalongan), R. M. A. Soerjoadmodjo (the Regent and Patih of Paku Alam Yogyakarta), R. Soerjodiprodjo (the Patih of Temanggung), Soetioso Sosro Boesono (the Wedana of Tegal), R. Soetirto (the Wedana of Prembun), R. T. Soetirto Pringo Haditirto (the Regent of Brebes), R. Sosrodiprodjo (the Patih of Purwokerto), R. T. A. Sosrodiprodjo (the Regent of Wonosobo), R. T. Sosrohadiwidjojo (the Regent of Volksraad, 1921–1931), R. M. A. A. Tjokro Adikoesoemo (the Regent of Temanggung), R. T. A. Tjondro Negoro (the Regent of Sidoarjo), R. A. A. Wiranatakoesoema (the Regent of Bandung in 1920–1931 and Cianjur in 1912–1920, and the member of Volksraad in 1922–1935), R. T. Wreksodiningrat (the Regent of Solo).

Besides in big colonial cities, Freemasonry also spread its understanding in *kring*, a gathering place smaller than a lodge. Several *kring* areas were Karawang, Cirebon, Purworedjo, Jember-Bondowoso, Ambon, Slamat, Indrapura, Lawang, Pontianak, etc (Arifin, 2018). The *kring* showed the development of the Freemasonry organization which infiltrated remote areas, besides big colonial cities, to develop secularism in social life and liberalism in religion.

The Regent of Karawang, R. Aria Soeriamihardja, had become a Freemasonry member since 1925. P. Lugt in the report of Krawang Kring activities stated that R. Aria Soeriamihardja was a speaker in a Freemasonry activity on March 21, 1937. His speech entitled “It was over de mystiek van den Islam,” which means Something about Mysticism in Islam. He delivered that the desire to know God revealed to him in Islam was necessary through several activities (Arifin, 2018):

1. Performing daily religious practices,
2. Acknowledging the essential truth,
3. Finding revelation through meditation and certain taboos,
4. Knowing the essence of truth, namely esoteric teachings.

Esoteric teachings are hidden religious education, which can only be understood by some groups, especially those initiated by certain associations (Wiryawan, 2014). In other words, these comprehensive teachings refer to secret teachings. This reason made the term esoteric is often associated with mysticism and the occult. Occultism constitutes the knowledge and study of supernatural, magical, and mysterious powers, and is associated with pagan practices. In this context, many Muslims, such as the Sarekat Islam and Muhammadiyah figures, did not agree with the teachings of Freemasonry because they disturbed the purity of Aqedah in Islamic teachings.

## 5 The accumulation and contest of the struggle of the Islamic ideology and colonialism freemasonry

In the grand meeting of the Syarikat Islam in Surabaya in 1913, H. Oemar Said Tjokroaminoto raised awareness of Muslims to organize and promote unity and grow their political power before the Dutch Colonists. They could only attain victory with a united force. The victory would enable Muslims to gain their independence. The meeting also raised the Muslims' awareness of the importance of a nation's independence. Political independence would release Muslims from the shackles of colonialism and could create their destiny to gain prosperity and justice (Suryanegara, 2017). Moreover, Syarikat Islam immediately organized a demonstration by forming the Army of Kanjeng Nabi Muhammad to reject the insulting newspaper writings published by Djawa Hisworo Magazine in Surakarta on January 11, 1918, and entitled “Pertjakapan Marto and Djojo” and “Goesti Kanjeng the Prophet Rasul drank wine and opium” (Agung, 2016a, Agung, 2016b). The case became big because it was discussed in the People's Council (Volksraad) chaired by MGJ Bisschop, and most members were from Boedi Oetomo. Hisworo Magazine was likely a part of a Freemasonry conspiracy arising from Javanese aristocrats, who dared to insult Islam. On January 30, 1918, Syarikat Islam, represented by Abikusno Tjokrosujono (HOS. Cokroaminoto's younger brother), wrote on the SI newspaper, Oetoesan Indies, to demand the punishment of Marthodarsono as a government author of Susuhunan Pakubuwono X (Affandie, 2017).

Persyarikatan Muhammadiyah was established as an organization in 1912 to answer the collapse of human values due to the Dutch colonial policy on Forced Cultivation (1830–1912). This policy resulted in poverty, ignorance and poor public health, destruction of religious beliefs, and many orphaned children due to misery. KH. Ahmad Dahlan and his wife, Mrs. Aisiyah, carried out a social propaganda reform movement by establishing orphanages, building schools and health houses, and encouraging capable people to contribute and donate in the *fastabiqulkahiraat* forum. The jargon of returning to the Qur'an and Al-hadith teachings was a form of Islamic ideological resistance against secularism, feudalism, and colonialism (Deliar, 1980).

Meanwhile, KH. Hasyim Asy'ari with other scholars initiated the establishment of the Nahdlatul Ulama movement to oppose colonialism responded to the Wahhabism movement dividing Muslims into several groups. Wahhabism was also allegedly carried by the colonialists. Besides awakening the ulama to unite, Nahdlatul Ulama reestablished *Ahl Sunnah wal Jama'ah* as an Islamic creed instilled by the Wali Sanga as the Islamic propagators in the archipelago (Abdul and Adnan, 2012). The ulema movement fortified Muslims in various *pesantren*, especially in multiple villages to oppose thoughts influenced by the colonial accumulation. Nahdlatul Ulama spread the understanding of Aswaja through Tashwirul Afkar magazine and its youth and women organizations, such as Ansor, Banser, Fatayat, and Muslimat. The results of deliberations and various thoughts of the ulama's were disseminated in the NU organizational bodies in the regional levels, branch levels, and various villages. The NU *santri* movement reflected Islam and nationality from various ideologies.

There were still many Indonesian Islamic ideological movements emerging as a result of colonialism. Three Islamic movements, including Syarikat Islam, Muhammadiyah, and Nahdlatul Ulama, represented several patterns and forms of responses to colonialism.

## 6 Conclusion

Freemasonry carried a secular ideology that rejected all forms of religious intervention in human life. Therefore, their criticisms were directed to religions, especially Islam, the biggest religion in Java. The secular ideology infiltrated by indigenous elites led Freemasonry to resist Islamic movements, such as Syarikat Islam, Muhammadiyah, Nahdlatul Ulama, Persis, Al-Irsyad, etc. that revived against colonialism and Western colonialism. The Dutch Colonial Government used Freemasonry and indigenous elites, who joined forces to confront these Islamic movements and solve the ideological conflicts. Freemasonry attempted to clash Javanese culture with Arabic (Islamic) culture and separate indigenous elites from religious groups endangering colonialism. These interests intersected with Freemasonry's goals of spreading fanatical views against formal religious formulations, rejecting various forms of religious fanaticism, and fighting dogma (belief). Indoctrination of Freemasonry teachings, such as liberalism and secularism, was conducted massively through lectures, discussions, member meetings, social activities, and mass media. These common interests led to a mutually beneficial relationship between the colonial practices and Freemasonry roles in dealing with religious movements against colonialism.

## Declarations

### Author contribution statement

Ajid Thohir: Conceived and designed the experiments; Performed the experiments; Analyzed and interpreted the data; Wrote the paper.

Dedi Supriadi: Performed the experiments; Analyzed and interpreted the data;

Mulyana: Conceived and designed the experiments; Analyzed and interpreted the data.

Faizal Arifin, Muhammad Andi Septiadi: Analyzed and interpreted the data; Contributed reagents, materials, analysis tools or data.

### Funding statement

This work was supported by UIN Sunan Gunung Djati Bandung.

### Data availability statement

Data will be made available on request.

### Declaration of interests statement

The authors declare no conflict of interest.

### Additional information

No additional information is available for this paper.
